# Choosing the optimal immunotherapeutic strategies for non-small cell lung cancer based on clinical factors

**DOI:** 10.3389/fonc.2022.952393

**Published:** 2022-08-12

**Authors:** Natsuki Nakagawa, Masanori Kawakami

**Affiliations:** Department of Respiratory Medicine, The University of Tokyo Hospital, Tokyo, Japan

**Keywords:** aged, interstitial lung disease (ILD), liver metastasis, performance status (PS), pleural effusion

## Abstract

The treatment landscape of advanced non-small cell lung cancer (NSCLC) has changed dramatically since the emergence of immune checkpoint inhibitors (ICIs). Although some patients achieve long survival with relatively mild toxicities, not all patients experience such benefits from ICI treatment. There are several ways to use ICIs in NSCLC patients, including monotherapy, combination immunotherapy, and combination chemoimmunotherapy. Decision-making in the selection of an ICI treatment regimen for NSCLC is complicated partly because of the absence of head-to-head prospective comparisons. Programmed death-ligand 1 (PD-L1) expression is currently considered a standard biomarker for predicting the efficacy of ICIs, although some limitations exist. In addition to the PD-L1 tumor proportion score, many other clinical factors should also be considered to determine the optimal treatment strategy for each patient, including age, performance status, histological subtypes, comorbidities, status of oncogenic driver mutation, and metastatic sites. Nevertheless, evidence of the efficacy and safety of ICIs with some specific conditions of these factors is insufficient. Indeed, patients with poor performance status, oncogenic driver mutations, or interstitial lung disease have frequently been set as ineligible in randomized clinical trials of NSCLC. ICI use in these patients is controversial and remains to be discussed. It is important to select patients for whom ICIs can benefit the most from these populations. In this article, we review previous reports of clinical trials or experience in using ICIs in NSCLC, focusing on several clinical factors that are associated with treatment outcomes, and then discuss the optimal ICI treatment strategies for NSCLC.

## 1 Introduction

In the last 10 years, immune-checkpoint inhibitors (ICIs) have changed treatment strategies for non-small cell lung cancer (NSCLC). The benefit of ICIs over previous standard therapy (cytotoxic chemotherapy) has been demonstrated both as monotherapy and as combination therapy, regardless of previous treatment history (1–7). Response duration of ICIs tends to be longer than cytotoxic chemotherapy (1, 2, 5, 6). Survival duration of some patients with advanced NSCLC treated with ICI exceeded 3 years and notably, the 5-years follow-up form KEYNOTE-024 shows an OS rate of 32% (6, 8–13). In clinical trials, the 2-year survival rate of advanced NSCLC patients is 37%–45% when treated with combination therapy of ICI and chemotherapy as the first-line treatment and 23%–29% with ICI monotherapy for previously treated patients, while 18%–29% in treatment-naive patients and 8%–16% in previously-treated patients when treated with chemotherapy (14–16). Despite of these improved treatment outcomes by ICIs in clinical trials, there are still issues to be addressed in daily clinical practice. First, it is difficult to determine which treatment regimen is most suitable for individual cases. Many treatment regimens are available for patients with advanced NSCLC. Most patients with NSCLC experience disease progression as a result of primary or acquired resistance to ICIs (17, 18). In this review, we discuss the clinical factors that could influence the efficacy and safety of drugs including ICIs. Second, many patients in clinical practice do not fulfil the eligibility criteria for clinical trials (19–23). For example, aged patients or patients with poor performance status (PS) are usually considered ineligible for prospective clinical trials. Generally, because of their poor condition, it is difficult to treat these patients with cytotoxic chemotherapy, and their prognosis is worse than that of patients who fulfill the eligibility criteria of clinical trials. ICIs have different toxicity profiles than standard chemotherapy, and their cytotoxicity is usually mild. Therefore, ICIs may be a good treatment option for patients who do not meet the criteria for chemotherapy. It is important to select patients for whom ICIs can benefit the most from this population. In this review, we will summarize previous clinical studies of ICIs used for NSCLC, and then discuss the optimal ICI treatment strategies, focusing on the clinical factors that potentially predict ICI effects.

## 2 Previous randomized control trials including ICIs for NSCLC

Many studies on ICIs have been conducted in patients with advanced or recurrent NSCLC. **Table 1** shows the major clinical trials that tested ICI regimens for treatment-naïve patients in which the primary endpoints were positive and negative, respectively. **Tables 1A**, **C** are for squamous NSCLC (Sq-NSCLC), while **Tables 1B**, **D** represent non-squamous NSCLC (NSq-NSCLC). The efficacy results were almost the same between Sq and non-Sq patients, except for the KEYNOTE-024 study. It should be noted that the results of KEYNOTE-024, KEYNOTE-042, IMPOWER-110, and KEYNOTE-598 includes both squamous and non-squamous NSCLC patients.

**Table 1:** Key clinical trials that tested ICI regimens for treatment-naïve patient.

A. Sq-NSCLC, positive study.							
Study name	PD-L1 expression	experimental arm	control arm	OS (months, 95% CI)	OS HR [95% CI]	PFS (months, 95% CI)	PFS HR [95% CI]	ORR
KN407 (16, 24)	All	Pembro + Chemo	Placebo + Chemo	17.1 [14.4–19.9]	0.71 [0.58–0.88]	8.0 [6.3–8.4]	0.57 [0.47–0.69]	62.6%
KN024 † (2, 8)	≥ 50%	Pembro	Chemo	26.3 [18.3–40.4]	0.62 [0.48–0.81]	7.7 [6.1–10.2]	0.50 [0.39–0.55]	46.1%
KN042 † (25)	≥ 1%	Pembro	Chemo	16.7 [13.9–19.7]	0.81 [0.71–0.93]	5.4 [4.3–6.2]	1.07 [0.94–1.21]	27%
IM110 † (5, 11)	TC/IC 3 §	Atezo	Chemo	20.2 [17.2–25.6]	0.83 [0.62–1.10]	8.2 [6.8–11.4]	0.59 [0.43–0.81]	40.2%
CM227 ‡ (6, 13)	≥ 1%	Nivo + Ipi	Chemo	15.0 [12.5–18.7] ¶	0.63 [0.49–0.79]	4.1 [2.9–5.6]	0.77 [0.57–1.05]	34.7%
	negative	Nivo + Ipi	Chemo	NA	NA	5.1 [3.5–6.4]	0.74 [0.58–0.94]	27.3%
CM9LA ‡ (7, 10)	All	Nivo + Ipi + Chemo	Chemo	14.5 [13.1–19.3]	0.63 [0.47–0.85]	5.6 [4.3–9.7]	0.60 [0.44–0.81]	48.7%
EMP-L1‡ (26)	≥ 50%	Cemip	Chemo	NA	0.53 [0.36–0.77]	NA	0.53 [0.40–0.70]	NA

B. Nsq-NSCLC, positive study.							
KN024 † (2, 8)	≥ 50%	Pembro	Chemo	26.3 [18.3–40.4]	0.62 [0.48–0.81]	7.7 [6.1–10.2]	0.50 [0.39–0.55]	46.1%
KN042 † (25)	≥ 1%	Pembro	Chemo	16.7 [13.9–19.7]	0.81 [0.71–0.93]	5.4 [4.3–6.2]	1.07 [0.94–1.21]	27%
KN189 (12, 14, 27)	All	Pembro + Chemo	Placebo + Chemo	22.0 [19.5–24.5]	0.56 [0.46–0.69]	9.0 [8.1–10.4]	0.49 [0.41–0.59]	48.3%
CM227 ‡ (6, 13)	≥ 1%	Nivo + Ipi	Chemo	19.2 [15.7–21.7] ¶	0.77 [0.66–0.90]	5.5 [4.1–7.6]	0.83 [0.68–1.01]	37.1%
	negative	Nivo + Ipi	Chemo	NA	NA	4.3 [2.9–6.4]	0.81 [0.62–1.07]	24.1%
CM9LA‡ (7, 10)	All	Nivo + Ipi + Chemo	Chemo	17.8 [14.1–20.7]	0.78 [0.63–0.96]	7.0 [5.6–8.3]	0.72 [0.59–0.88]	32.9%
IM150 (4, 9)	All	Atezo + Bev + Chemo	Bev + Chemo	19.5 [17.0–22.2]	0.80 [0.67–0.95]	8.4	0.57 [0.48–0.67]	63.5%
IM130 (28)	All	Atezo + Chemo	Chemo	18.6 [16.0–21.2]	0.79 [0.64–0.98]	7.0 [6.2–7.3]	0.64 [0.54–0.77]	49.2%
IM110 † (5, 11)	TC/IC 3 §	Atezo	Chemo	20.2 [17.2–25.6]	0.83 [0.62–1.10]	8.2 [6.8–11.4]	0.59 [0.43–0.81]	40.2%
EMP-L1 ‡ (26)	≥ 50%	Cemip	Chemo	NA	0.83 [0.59–1.16]	NA	0.65 [0.51–0.84]	NA
TASUKI-52 (29)	All	Nivo + Bev + Chemo	Bev + Chemo	25.4 [21.8–NR]	0.85 [0.63–1.14]	12.1 [9.8–14.0]	0.56 [0.43–0.71]	61.5%

† Both squamous and non-squamous histology are included, ‡ Subgroup analysis based on the histology, § This study met the primary outcome only in a TC/IC 3 population at first analysis, ¶ Histology-based OS was analyzed in the PD-L1 expression ≥1% and <1% combined patient population.

PD-L1, Programmed death-ligand 1; OS, overall survival; CI, confidence interval; PFS, progression free survival; ORR, objective response rate; NA, not available; NR, not reached.

**Table 1:** Key clinical trials that tested ICI regimens for treatment-naïve patient.

C. Sq-NSCLC, negative study.							
Study name	PD-L1 expression	experimental arm	control arm	OS (months, 95% CI)	OS HR [95% CI]	PFS (months, 95% CI)	PFS HR [95% CI]	ORR
IM131 (50)	All	Atezo + Chemo	Chemo	14.2 [12.3–16.8]	0.88 [0.73–1.05]	6.3 [5.7–7.1]	0.71 [0.60–0.85]	49.7%
CM026 ‡ (30)	≥ 5%	Nivo	Chemo	10.9 [NA]	0.77 [0.48–1.25]	3.3 [NA]	0.87 [0.53–1.41]	NA
Govidant et al. (53)	≥ 1%	Ipi + Chemo	Placebo + Chemo	13.4 [11.8–14.8]	0.91 [0.77–1.07]	5.6 [5.4–5.9]	0.87 [0.75–1.01]	44%
MYSTIC (31) †	≥ 25%	Durva	Chemo	16.3 [12.2–20.8]	0.76 [0.56–1.02]	4.7 [3.1–6.3]	0.87 [0.59–1.29]	35.6%
		Durva + Treme	Chemo	11.9 [9.0–17.7]	0.85 [0.61–1.17]	3.9 [2.8–5.0]	1.05 [0.72–1.53]	34.4%
KN598 (32) †	≥ 50%	Ipi + Pembro	Placebo + Pembro	21.4 [16.6–NR]	1.08 [0.85–1.37]	8.2 [6.0–10.5]	1.06 [0.86–1.30]	45.4%

D. Nsq-NSCLC, negative study.							
CM026 ‡ (30)	≥ 5%	Nivo	Chemo	14.5 [NA]	1.13 [0.84–1.50]	4.2 [NA]	1.24 [0.95–1.62]	NA
MYSTIC (31) †	≥ 25%	Durva	Chemo	16.3 [12.2–20.8]	0.76 [0.56–1.02]	4.7 [3.1–6.3]	0.87 [0.59–1.29]	35.6%
		Durva + Treme	Chemo	11.9 [9.0–17.7]	0.85 [0.61–1.17]	3.9 [2.8–5.0]	1.05 [0.72–1.53]	34.4%
KN598 (32) †	≥ 50%	Ipi + Pembro	Placebo + Pembro	21.4 [16.6–NR]	1.08 [0.85–1.37]	8.2 [6.0–10.5]	1.06 [0.86–1.30]	45.4%
IM132 (33)	All	Atezo + Chemo	Chemo	17.5 [13.2–19.6]	0.86 [0.71–1.06]	7.6 [6.6–8.5]	0.60 [0.49–0.72]	47%

† Both squamous and non-squamous histology are included, ‡ Subgroup analysis based on the histology, § This study met the primary outcome only in a TC/IC 3 population at first analysis, ¶ Histology-based OS was analyzed in the PD-L1 expression ≥1% and <1% combined patient population.

PD-L1, Programmed death-ligand 1; OS, overall survival; CI, confidence interval; PFS, progression free survival; ORR, objective response rate; NA, not available; NR, not reached.

## 3 Clinical predictive factors for ICI treatment outcomes

### 3.1 Programmed death-ligand 1 (PD-L1) expression

PD-L1 tumor proportion score (TPS) is widely used to predict ICI effects. In the phase 2 KEYNOTE-001 trial, the objective response rates (ORR) of pembrolizumab for pre-treated NCSLC patients were 45%, 17%, and 11% in the subgroup with PD-L1 TPS score, assessed by the PD-L1 IHC 22C3 pharmDx, with ≥50%, 1%–49%, and <1%, respectively (34). Furthermore, the survival benefit of pembrolizumab was also associated with a high PD-L1 TPS. The superiority of pembrolizumab monotherapy over chemotherapy for treatment-naïve NSCLC patients was observed in both the PD-L1 TPS ≥ 50% and ≥1% groups in KEYNOTE-024 and 042 (2, 25). Subgroup analysis of these studies showed the association of higher PD-L1 TPS with better efficacy of pembrolizumab. This association was confirmed in real-world settings when limited to PD-L1 TPS ≥ 50% (35–37). These data support the notion that PD-L1 TPS assessed by 22C3 assay predicts the outcome of pembrolizumab monotherapy used in the first-line setting. Similar trends have been observed in clinical trials for other cancers (38–40).

The association between PD-L1 TPS and ICI effects was inconsistent when different methods were used to evaluate the TPS. In the Impower110 trial, the efficacy of atezolizumab monotherapy used as a first-line treatment in NSCLC patients was correlated with PD-L1 TPS assessed by SP142 assay, and superiority of atezolizumab over platinum-based chemotherapy was observed in the subgroup with PD-L1 TPS ≥ 50% (5). However, in the CheckMate 026 trial where nivolumab efficacy was tested, no superiority of nivolumab over platinum-based chemotherapy was seen either in the pre-planned group with PD-L1 TPS ≥ 5% or in the exploratory subgroup with PD-L1 TPS ≥ 50% (30). In this study, the PD-L1 TPS was assessed using the 28-8 antibody. This inconsistency among the studies may be attributed from the fact that the assessment assay used to evaluate PD-L1 expression differed in each clinical trial. On the other hand, Impower 110 trial compared PD-L1 scoring methods, SP142, 22C3 and SP263, as an exploratory analysis. Of note, median OS among patients with high PD-L1 TPS assessed by these three assays were similar. In clinical trials, PD-L1 assays often differ among different ICI drugs. Few information is available concerning analysis of the concordance among different PD-L1 assays (41).

In addition to the methods for TPS evaluation, the cut-off levels of PD-L1 expression are not fixed, and they sometimes change even in the middle of ongoing clinical trials (42–44). Another factor that may lead to these inconsistent results is heterogeneity of PD-L1 expression. The spatial and temporal heterogeneity of PD-L1 expression in the same tumor has been previously reported (45–49). PD-L1 expression tends to be high in primary sites, adrenal glands, liver, and lymph nodes, but low in the bone and brain (45, 46). When PD-L1 TPS of lymph node metastatic site was assessed, the association with ICI efficacy was not observed (45). In the clinical trials discussed above, the number of biopsy sites where PD-L1 TPS was evaluated varied among cases.

Inconsistency in the predictive value of PD-L1 expression among clinical trials was also observed in the setting of combination chemoimmunotherapy. PD-L1 expression was positively correlated with progression-free survival (PFS) in the combination of pembrolizumab with platinum plus pemetrexed for NSq-NSCLC; atezolizumab with carboplatin, paclitaxel, and bevacizumab for NSq-NSCLC; and atezolizumab with carboplatin plus nab-paclitaxel for Sq-NSCLC (4, 27, 50). However, this correlation was not proven in the atezolizumab with carboplatin plus nab-paclitaxel combination for NSq-NSCLC and pembrolizumab with carboplatin plus either paclitaxel or nab-paclitaxel for Sq-NSCLC (24, 28). The aforementioned ICIs are inhibitors of the PD-1/PD-L1 checkpoint pathway. In contrast, ipilimumab is a monoclonal antibody for cytotoxic T-lymphocyte associated protein 4 (CTLA-4), which is independent of the PD-1/PD-L1 pathway. It is reasonable that this agent can be effective even in PD-L1-negative population (6, 13). In fact, in the CheckMate 9LA trial, where the combination of nivolumab and ipilimumab with chemotherapy was studied, favorable outcomes were observed regardless of PD-L1 expression for both NSq-NSCLC and Sq-NSCLC (7, 10). This trend is consistent with previous clinical trials involving patients with melanoma and renal-cell carcinoma (51, 52). However, when nivolumab and ipilimumab are used without chemotherapy for patients with NSCLC, median overall survival (OS) was numerically greater in higher PD-L1 expression population in the CheckMate 227 trial (13). Considering the negative result of the KEYNOTE-598 study, where pembrolizumab plus ipilimumab for metastatic NSCLC with PD-L1 TPS ≥ 50% was tested (**Tables 1C, D**), the benefit of adding ipilimumab to an anti PD-1 antibody for patients with PD-L1 TPS ≥ 50% should be discussed carefully (32).

Overall, although the results are inconsistent, PD-L1 expression can be used as a predictive biomarker for ICI effects. Recently, a combined positive score has emerged as a new method instead of PD-L1 TPS to evaluate PD-L1 expression (54). A combined positive score is calculated as the proportion of tumor cells, lymphocytes, and macrophages that were positively stained by PD-L1 immunohistochemical staining of total tumor cells. The KEYNOTE-048 trial of pembrolizumab treatment for head and neck cancer demonstrated a positive association of favorable survival with PD-L1 expression level assessed by the combined positive score (43).

### 3.2 Driver mutation

A correlation between driver mutation subtypes and ICI efficacy has been reported. The ImmunoTarget group retrospectively compared ORR after ICI treatment among NSCLC patients with various driver mutations. It was revealed that the KRAS-driven and BRAF-driven subgroups appreciated a greater benefit from ICI than EGFR-driven or ALK-driven subgroups (55).

Several clinical trials have suggested favorable efficacy of ICIs in NSCLC patients with KRAS mutations (1, 56–60). When pembrolizumab was used as monotherapy in NSq-NSCLC patients with PD-L1 TPS ≥ 50%, KRAS mutation was associated with longer OS, while this association was not observed when pembrolizumab was used as combined chemoimmunotherapy (61, 62). Notably, co-mutation of SKT11/LKB11 with KRAS mutation, which exists in approximately 30% of KRAS-mutated NSCLC, is associated with an unfavorable efficacy of ICIs (63, 64). This mutation was associated with lower PD-L1 expression and fewer tumoricidal immune infiltrates.

Many recent clinical trials of ICIs have excluded those with actionable driver mutations, such as EGFR mutations and ALK fusions. The decision for this exclusion is probably based on the results of a subgroup analysis in large randomized controlled trials conducted in the early days of the ICI era, such as CheckMate 017, CheckMate 057, KEYNOTE-010, and OAK, which compared ICIs and docetaxel for their efficacy and safety as second-line therapy in advanced NSCLC (1, 56, 65, 66). The meta-analysis of these trials demonstrated that the integrated OS hazard ratio of ICIs compared to docetaxel was 1.05 [95% confidence interval [CI]: 0.70–1.55] in the EGFR-mutant subgroup and 0.66 [95% CI: 0.58–0.76] in the EGFR wild-type subgroup (59, 60, 67). Retrospective studies have also shown generally low efficacy of ICIs in driver mutation-positive NSCLC (55, 68, 69). In addition, a recently published phase 2 study comparing nivolumab and carboplatin-pemetrexed for EGFR-mutated NSCLC with resistance to EGFR-tyrosine kinase inhibitors (TKI) revealed significantly worse survival in patients treated with nivolumab (70). Poor efficacy of ICIs for NSCLC patients with EGFR mutations is thought to be derived from a lower tumor mutation burden and an immunosuppressive tumor microenvironment (71, 72).

However, some prospective studies have shown comparable or superior efficacy of ICIs in NSCLC patients with driver mutations (73–77). An exploratory subgroup analysis of the IMpower150 trial demonstrated that in NSq-NSCLC patients with sensitizing EGFR mutations, OS of atezolizumab, carboplatin, paclitaxel, and bevacizumab combination group was longer than carboplatin, paclitaxel, and bevacizumab combination group. (median OS was26.1 months [95% CI 17.0–41.4] in the atezolizumab arm *vs.* 20.3 months [95% CI 13.4–33.6] in the control arm; hazard ratio [HR] 0.91 [95% CI 0.53–1.59]) (73, 74). Based on these results, two prospective studies are ongoing in Japan to verify the efficacy and safety of combination chemoimmunotherapy with atezolizumab, carboplatin, paclitaxel, and bevacizumab in EGFR-mutant NSCLC patients who were already treated with an EGFR-TKI (78, 79).

Usually, molecular-targeted therapies are more effective than ICIs or cytotoxic agents for NSCLC patients with actionable driver mutations (78–88). Combination therapy with TKIs and ICIs has failed due to severe adverse events (89–91). Based on the idea of “best drug first,” there is no doubt that the first-line therapies for NSCLC patients with actionable driver mutations are TKIs (92–94). However, to the best of our knowledge, there is no clear conclusion as to whether ICIs can be a treatment option for these patients at any late treatment line. Some retrospective studies have suggested that PD-L1 expression predicts ICI efficacy, even in EGFR-mutant NSCLC (95). Furthermore, interestingly, PD-L1 expression was upregulated after EGFR-TKI therapy *via* ERK1/2 pathway modulation (47, 48). It has also been reported that EGFR mutations can upregulate PD-L1 expression through the Ras/RAF/MEK/ERK, PI3K/AKT/mTOR, JAK/STAT, NF-kB, and YAP pathways (96–99). Further studies are warranted to clarify the association of driver mutations with PD-L1 expression or ICI efficacies.

### 3.3 Metastatic site

#### 3.3.1 Liver

Liver metastases have been validated as negative prognostic factors for NSCLC patients (100, 101). More metastases in the liver are correlated with worse survival (102). In addition, the presence of liver metastases predicts poor outcomes after ICI monotherapy (3, 36, 72, 103–106). One possible underlying mechanism is systemic immune tolerance which is mediated by a number of specialized antigen-presenting cells, including dendritic cells, Kupffer cells, liver sinusoidal endothelial cells, and hepatic stellate cells (102, 105, 107–110). From the viewpoint of PD-L1 spatial heterogeneity, PD-L1 expression was relatively higher in liver than other organs in NSCLC patients (45). Conversely, the presence of liver metastases was associated with a lower CD8+ T-cell count at the invasive tumor margin among patients with NSCLC and melanoma who received pembrolizumab (105). This suggests systemic activation of the regulatory immune microenvironment in patients with liver metastases, which results in a poor response to ICI treatments in the presence of liver metastases despite the relatively higher PD-L1 expression.

Currently, there is no consensus regarding the optimal treatment for NSCLC with liver metastases. Although cytotoxic agents and ICIs elicit relatively little efficacy in NSCLC with liver metastases when used alone, one retrospective study showed that combination chemotherapy may be effective (111). Some clinical trials have also suggested that the addition of bevacizumab to ICI treatment is effective for patients with NSCLC with liver metastases (29, 73, 112). Bevacizumab is an anti-vascular endothelial growth factor (VEGF) antibody. Preclinical and clinical data have demonstrated that bevacizumab normalizes vasculature, restores dendritic cell maturation, and reduces T-regulatory cells and myeloid-derived suppressor cells in cancer patients (113–117). Considering these pharmacological effects, treatment regimen containing bevacizumab may be reasonable for patients of NSCLC with liver metastases, where immunosuppressive microenvironment is an issue for ICI treatments as discussed above.

The presence of liver metastases is thought to be associated with the onset of hyperprogressive disease (HPD) (118–120). HPD is characterized by rapid disease progression after initiation of ICIs, often defined as a > 50% increase in tumor size within less than 2 months after initiation of ICIs, although currently there is no widely accepted definition (118, 121). HPD is associated with worse clinical outcomes (118). Other than liver metastases, high LDH levels, low Albumin levels, multiple metastatic sites, poor PS, and a Royal Marsden Hospital prognostic score of ≥ 2 were associated with the risk of HPD occurrence (118, 120). However, underlying mechanisms of HPD are not understood well. Treatment strategies for NSCLC patients at high risk of HPD have not yet been established.

#### 3.3.2 Brain

Radiation therapy is the most important treatment strategy that should be considered first for NSCLC patients with brain metastases (BMs), especially when clinical symptoms derived from BMs are present (122). Thus the role of ICIs, with or without cytotoxic agents, can be discussed only for the regulation of BMs that are asymptomatic or already treated with radiation. The efficacy of ICI in patients with leptomeningeal disease requires further investigation (123).

The survival benefit of ICIs is similar regardless of the presence or absence of BMs based on a subgroup analysis of clinical trials of ICIs with or without cytotoxic agents, as listed in Table 1 (6, 10, 12, 26, 29, 124, 125). A meta-analysis of 10 clinical trials with ICIs in NSCLC showed an OS HR of 1.25 (95% CI = 1.09–1.44, I2 = 43.8%, P <.001) for BMs compared with those without BMs (104). A retrospective study showed that the presence of BMs and a larger maximum diameter of brain metastases were associated with worse prognosis of NSCLC patients after ICI monotherapy in the second or later treatment line (126). To our knowledge, there are few available data regarding intracranial response rates to ICIs in NSCLC patients. Phase 2 studies on melanoma demonstrated that combination therapy with nivolumab and ipilimumab achieved higher intracranial response rates than treatment with nivolumab alone (127). Considering these data, patients with BMs can be treated in the same way as those without BMs, but combination immunotherapy with anti-PD-1 and anti-CTLA-4 agents, with or without cytotoxic agents, may provide better outcomes (124, 128, 129).

#### 3.3.3 Pleural effusion

Previous studies have reported that malignant pleural effusion is present in 11%–32% of patients with advanced NSCLC (130–132). Even a small amount of pleural effusion (< 10-mm thick on chest computed tomography) is an independent predictor of worse survival (130). This tendency was also observed in cases treated with ICIs, although the available data are limited to retrospective studies. The presence of malignant pleural effusion was associated with worse prognosis in NSCLC patients treated with a single ICI in either first-line or later treatment lines (126, 133–135). Recently reported retrospective study suggests that combination chemoimmunotherapy is more effective than pembrolizumab monotherapy as a first line treatment for NSCLC patients with malignant pleural effusion (136). As observed in liver metastases, malignant pleural effusion induces systemic immunosuppressive microenvironment through several mediators and pathways, including myeloid derived suppressor cells, neutrophils, macrophages, T-regulatory cells, and dysfunctional T cells that might result in low efficacy of ICIs (126, 137). As for safety, existence of pleural effusion before treatment with nivolumab was indicative of poor outcomes of interstitial lung disease (ILD) induced as an immune-related adverse event (irAE) when it occurs (138).

In the setting of combination chemoimmunotherapy, we could not find any studies to assess the effects of the presence of malignant pleural effusion on the efficacy of therapy or to evaluate which combination of drugs is better for use in cases with malignant pleural effusion. VEGF is thought to be one of the key factors that cause malignant plural effusion by increasing vascular and mesothelial permeability and capillary fluid leakage (139). In fact, several studies suggest the efficacy of bevacizumab for the management of malignant pleural effusion in Nsq-NSCLC (117, 140–144). VEGF also plays a principal role in immunosuppressive microenvironment as mentioned in the previous section (113–117). Therefore, the combination of bevacizumab and ICI is potentially a good treatment strategy for patients with malignant pleural effusion, although there is few evidence to support this, thus far.

## 4 ICIs for the special population

### 4.1 Elderly

An FDA meta-analysis of four randomized control trials in which ICI monotherapy and docetaxel were compared for patients with disease progression after platinum doublet treatment demonstrated similar survival benefits between these regimens, regardless of age (145). Another meta-analysis of clinical trials for other tumor types also showed a comparable efficacy of ICI monotherapy between patients aged ≥ 65 and < 65 years (19, 146). Furthermore, real-world data supported the evidence for efficacy and safety of ICI monotherapy for the elderly NSCLC patients (147–150). These data suggested that it is the PS or comorbidities rather than age that is associated with the outcome of ICI treatment in the elderly patients (19, 147, 148). It should be noted that the cutoff value for defining elderly varies among studies.

In combination chemoimmunotherapy, more attention should be paid to elderly patients. In the KEYNOTE-189 trials, in which treatment-naïve NSCLC patients were treated with a platinum agent and pemetrexed with or without pembrolizumab, the addition of pembrolizumab was associated with worse survival benefit in the elderly, which was defined as ≥ 75 years old (151). A retrospective study showed a similar result, that is, poor outcome of combination chemoimmunotherapy in the elderly group (152). In general, organ function declines with age, but it is difficult to evaluate these functions sufficiently with clinical examinations that are currently available. Clinical assessment tools for the elderly, such as the comprehensive geriatric assessment and Charlson comorbidity index, have been tested to predict the prognosis of anti-cancer therapy in many clinical trials, but their usefulness has not yet been established (153–157). Currently, there are no clinical assessment tools available to predict which elderly patients can tolerate chemoimmunotherapy well.

### 4.2 Performance status 2

In the ECOG 1594 study, which revealed almost similar efficacy and safety profiles among four platinum doublet regimens, a subgroup analysis showed that adverse events increased, and prognosis worsened in patients with a PS of 2 compared to those with a PS of 0 or 1 (158, 159). Historically, this is a pivotal study. Thereafter, for more than 10 years, cytotoxic agent monotherapies have been standard therapies for patients with a PS of 2. On the other hand, the advantage of ICIs is their favorable toxicity profiles. Therefore, ICIs may be an alternative treatment option for this population. Many studies have suggested that PS is not associated with the frequency or severity of irAEs (39, 150, 160–162). For example, in CheckMate 153, a prospective study validating the safety of second-line treatment with nivolumab for NSCLC patients aged ≥ 70 years and with a PS of 2, the incidence rates of grades 3–5 and any grade adverse events were not increased in the population with a PS of 2 (9% and 29%, respectively) compared to the overall population, including a PS of 0–2 (6% and 37%, respectively), and toxicity profiles were comparable between these populations (150). The toxicities of ICIs seem to be acceptable for patients with poor PS.

Regarding prognosis, in both prospective and retrospective studies of ICI monotherapy for NSCLC, patients with a PS ≥ 2 who were treated with ICI monotherapy showed poor survival (150, 161–165). The hazard ratio of PFS ranged from 2.00 to 2.39 and OS ranged from 2.72 to 2.82 in patients with a PS ≥ 2 compared with a PS of 0 or 1. Studies on ICI monotherapy in a relatively large number of patients with a PS ≥ 2 are summarized in **Table 2**. Unlike PFS and OS, ORR results were inconsistent among the studies. As shown in **Table 2**, some studies have shown that the ORR of patients with a PS 2 was comparable to that of patients with a PS 0–1 after ICI monotherapy (162, 165). Poor PS of NSCLC patients may result from many different reasons, such as cancer burden, cancer progression rate, comorbidities unrelated to cancer, or a combination of these factors. The analysis of ICI efficacy in patients with a PS 2 based on the reasons for poor PS may help us better understand who is suitable for ICI treatment in this population.

**Table 2:** ICI for PS2.

Author/year	Trial name	Type of study	Number of patients whose PS > 1	Proportion of PS > 1/total	Treatment line	Drug	OS (months, 95% CI)	PFS (months, 95% CI)	ORR	Incidence of TRAE of grade3-5
Spigel DR, et al., 2019 (150)	CheckMate 153	prospective	128	9.0%	2nd or later	Nivolumab	4.0 [3.1–6.2]	NA	NA	9%
Felip E, et al., 2020 (161)	CheckMate 171	prospective	103	12.7%	2nd or later	Nivolumab	5.2 [3.0–7.6]	NA	1.6%	6.8%
Middleton G, et al., 2020 (162)	PePS2	prospective	60	100%	1st: 40% Subsequent: 60%	Pembrolizumab	9.8 [7.1–14.6]	4.4 [3.3–9.9]	27%	73%
Matsubara T, et al., 2021 (163)		retrospective	11	8.8%	1st or 2nd: 43.2% 3rd or later: 56.8%	Nivolumab or Pembrolizumab	NA	NA	9.1%	18.2%
Sehgal K, et al., 2021 (165)		retrospective	29	39.2%	1st: 72.4% Subsequent: 27.6%	Pembrolizumab	4.1 [2.1–6.9]	2.3 [1.8–4.8]	17.9%	17.2%

PS, performance status; OS, overall survival; CI, confidence interval; PFS, progression free survival; ORR, objective response rate; TRAE, treatment related adverse events.

### 4.3 Interstitial lung disease

Patients with ILD have been excluded from most randomized controlled trials in which ICIs are involved. However, in the real world, ILD is seen frequently (at a rate of 14%) in treatment-naïve patients with NSCLC (166). ILD is an independent risk factor for drug-induced lung injuries, including ICI-related injuries and is associated with poor survival in NSCLC patients treated with ICIs (167–169). Drug-induced lung injuries caused by ICIs are the most common irAE that lead to the discontinuation of ICIs and are associated with worse survival (65, 66, 170). ILD includes a very wide spectrum, and its radiological classification is complex. Radiological assessments of ILD are different, even among radiologists (171, 172). This makes it difficult to stratify the degree of risks of pre-existing ILD for ICI-induced lung toxicities.

Several clinical trials have assessed the efficacy and safety of ICIs in patients with ILD. The AMBITIOUS trial is a prospective study of atezolizumab in NSCLC patients with idiopathic, chronic fibrotic interstitial pneumonia whose %VC was 70% or larger. This study was discontinued early because of the high incidence of pneumonitis (29.4%) (173). In this study, pre-existing honeycomb lung was associated with a high risk of frequency and severity of pneumonitis (57.1% of patients with pre-existing honeycomb lung suffered from drug-induced pneumonitis with a grade greater than or equal to 3). The honeycomb lung has also been reported to be associated with cytotoxic chemotherapy-related exacerbation of ILD (174). Another prospective study to evaluate the efficacy and safety of nivolumab in NSCLC patients with mild idiopathic interstitial pneumonia demonstrated favorable efficacy and a tolerable safety profile, where two out of 18 patients developed grade 2 pneumonitis (175). In this study, patients with mild idiopathic, classified as radiological possible or inconsistent with usual interstitial pneumonia (UIP), were included only when their %VC was 80% or more. Therefore, patients with radiological UIP patterns were excluded. These studies imply that the presence or absence of a honeycomb lung is the principal factor in predicting the safety of ICI treatment.

ILD related to ICIs may occur even in patients without ILD at the initiation of ICI therapy. Several risk factors for the onset and severity of ICI-induced lung toxicities have been suggested, including the primary tumor site of the lung, ICI combination therapy rather than ICI monotherapy, PD-1 inhibitors compared with PD-L1 inhibitors or CTLA-4 inhibitors, and the presence of pleural effusion before treatment (137, 176–179).

## 5 Discussion and conclusion

ICIs are now indispensable agents for NSCLC treatment and contribute to the extension of survival in NSCLC patients. Considering their relatively mild toxicities, ICIs could provide an opportunity of treatment for patients who cannot tolerate treatment with cytotoxic agents, such as elderly or patients with poor PS. As discussed in this paper, many clinical factors may affect the efficacy and safety of ICI treatment. PD-L1 is currently considered a predictive biomarker of ICI treatment, but clinicians should keep in mind that this is not a perfect biomarker as mentioned above. Emerging biomarkers, including tumor mutational burden, neoantigen load, tumor-infiltrating lymphocytes, immune-regulatory mRNA expression and blood biomarkers, are reported as possibly predictive (180). Further studies are warranted in this area.

## Author contributions

This review was drafted by NN and critically revised by MK. All authors contributed to the article and approved the submitted version.

## Funding

This work was supported by JSPS KAKENHI Grant Number 21H02776.

## Conflict of interest

The authors declare that the research was conducted in the absence of any commercial or financial relationships that could be construed as a potential conflict of interest.

## Publisher’s note

All claims expressed in this article are solely those of the authors and do not necessarily represent those of their affiliated organizations, or those of the publisher, the editors and the reviewers. Any product that may be evaluated in this article, or claim that may be made by its manufacturer, is not guaranteed or endorsed by the publisher.
